# Exosomes That Have Different Cellular Origins Followed by the Impact They Have on Prostate Tumor Development in the Tumor Microenvironment

**DOI:** 10.1002/cnr2.70001

**Published:** 2024-09-04

**Authors:** Cong Huang, Jialong Zhang, Hongzhi Wang, Chaozhao Liang

**Affiliations:** ^1^ Department of Urology The First Affiliated Hospital of Anhui Medical University, Anhui Medical University Hefei China; ^2^ Institute of Urology, Anhui Medical University Hefei China; ^3^ Key Laboratory of Genitourinary Diseases Anhui Province Anhui Medical University Hefei China

**Keywords:** biomarkers, exosomes, intercellular communication, prostate cancer, prostate cancer cells, tumor microenvironment

## Abstract

**Background:**

Prostate cancer (PCa) is the most common urinary tumor with the highest incidence rate and the second among the leading causes of death worldwide for adult males. In the worldwide cancer incidence rate, PCa is on the increase. The cancerous cells in the prostate and cells in the microenvironment surrounding the tumor communicate through signal transduction, which is crucial for the development and spread of PCa.

**Recent findings:**

Exosomes are nanoscale vesicles released into body fluids by various cells that can aid intercellular communication by releasing nucleic acids and proteins. Exosomes published by different types of cells in the tumor microenvironment can have varying impacts on the proliferation and growth of tumor cells via various signaling pathways, modes of action, and secreted cytokines.

**Conclusion:**

The main purpose of this review is to describe the effects of different cell‐derived exosomes in the tumor microenvironment of PCa on the progression of tumor cells, as well as to summarize and discuss the prospects for the application of exosomes in the treatment and diagnosis of PCa.

## 1 Introduction

**FIGURE 1:**
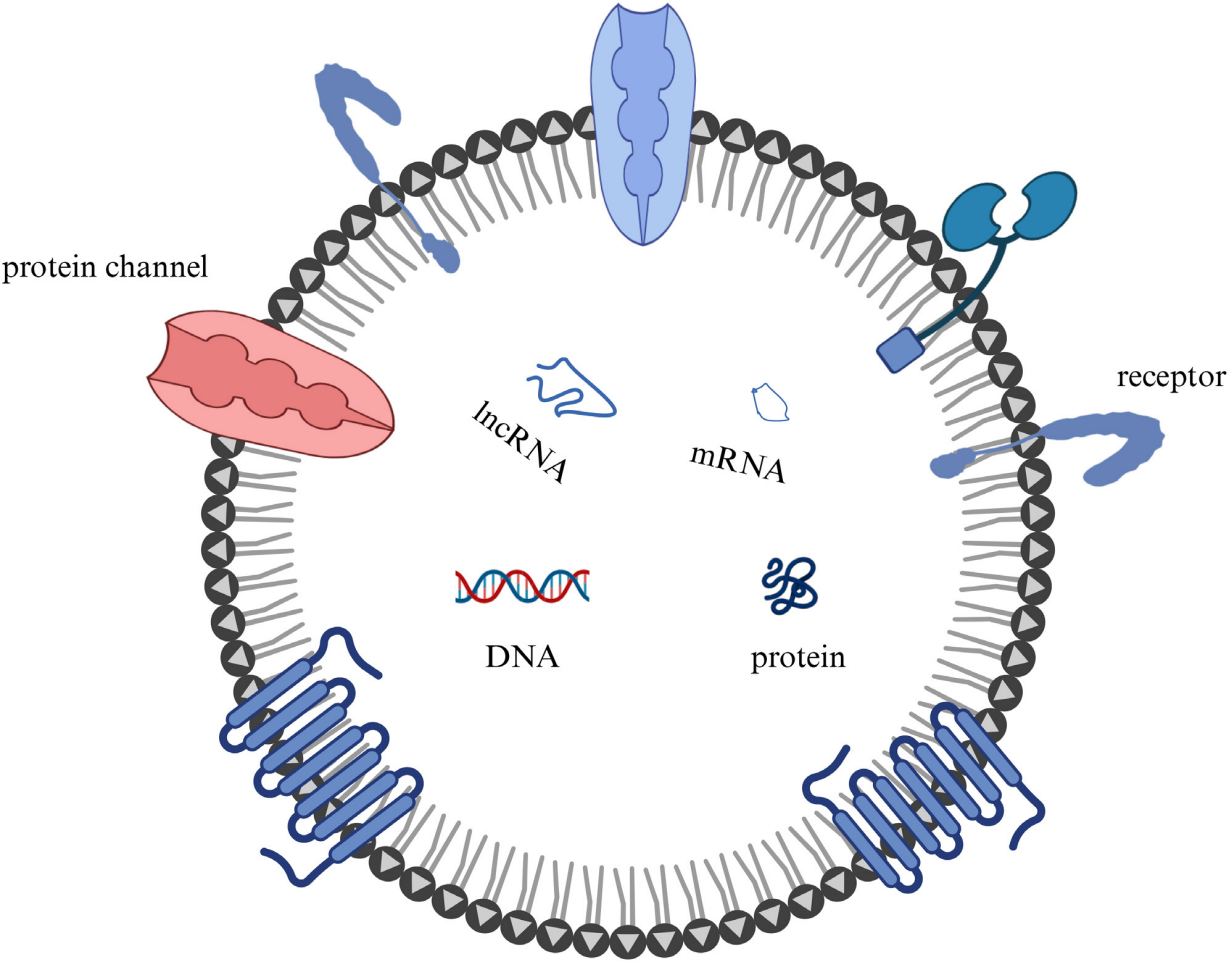
The fundamental structure of exosomes. Important information can be transported by exosomes, which also contain lipids, DNA, RNA, glycoconjugates, and a variety of proteins.

The prevalence rate of prostate cancer (PCa) is on the rise, making it the most common form of male‐specific malignancy and a major contributor to fatalities due to cancer [1, 2, 3, 4]. It is currently second only to lung cancer in terms of tumor prevalence [5]. Debatable is the specificity of PSA detection in clinical practice, yet early diagnosis and screening still maintain blood prostate‐specific antigen. As a result, PCa may be overdiagnosed and overtreated [6, 7, 8, 9]. Extracellular vesicles, or exosomes, have an average diameter of 100 nm and range in size from 30 to 200 nm [10, 11]. Exosomes are single‐membrane organelles that store a variety of components including nucleic acids, proteins, lipids, and glycoconjugates despite their tiny size (Figure 1). It is exactly because of its complex structure that it may function in tumor cells and be recognized by us. It could thus be utilized as a means of expediting the detection and assessment of the newest generation of tumor biomarkers in clinical practice. Exosomes are secreted in all cell types [12] and participate in various physiological and pathological processes [13, 14], this may be closely related to its structure. The function of extracellular and intercellular communication in the incidence and progression of PCa has slowly come to light in recent years. Exosomes also potential for the development of new therapeutic approaches due to their remarkable biophysical features, including high stability, high biocompatibility, and capacity to overcome biological barriers [15].

## 2 Exosomes Biogenesis

**FIGURE 2:**
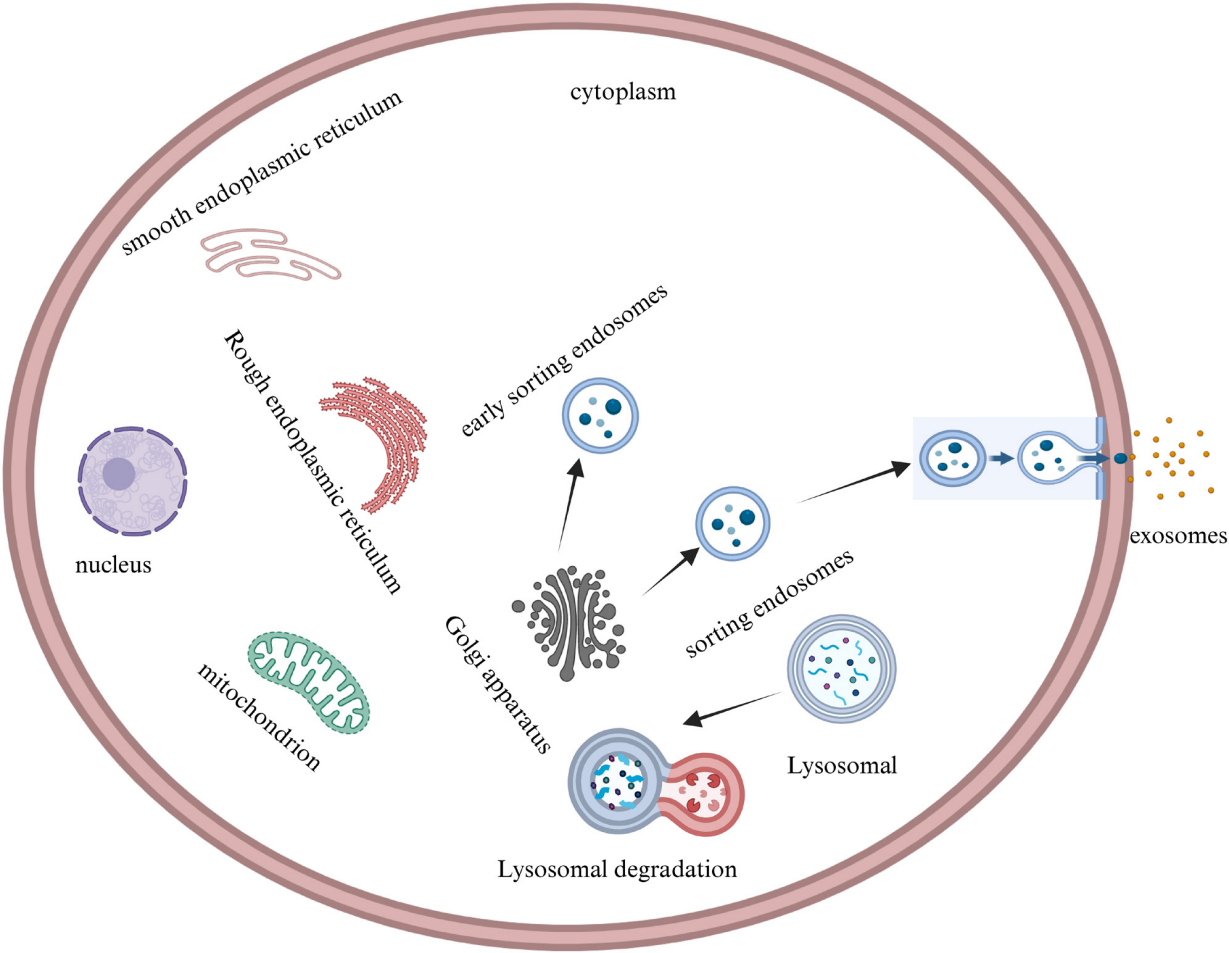
The exosomes' germinal process. Early sorting endosomes are formed by cellular membrane invagination, which is followed by the creation of multivesicle bodies. Lysosomes break down the residual material, while exocytosis allows the last portion to be expelled from the cell.

Nanoscale vesicles nicknamed exosomes are produced by endosomes. Therefore, the classic pathway of their biogenesis is the endosome pathway, which includes four main steps (Figure 2). First, different extracellular substances enter cells through endocytosis, and after coming into contact with the extracellular lipid membrane, foreign substances cause the production of proteins found on the surface (like CD9, CD63, and CD81) on the innermost component of the extracellular lipids membrane, which results in plasma membrane sprouting. Early sorting endosomes (ESEs) are created by some of these budding vesicles, while other ESEs are formed when the endoplasmic reticulum, the trans‐Golgi apparatus network (TGN), and mitochondrial components are regulated. Second, under the influence of TGN, the generated ESEs gradually develop into mature sorting endosomes. Third, the creation of multivesicular bodies (MVBs) is caused by the plasma membrane invaginating at numerous mature sorting sites. Fourth, some of the poly vesicles combine with autophagosomes or lysosomes, while the other part fuses with the plasma membrane. The substance produced by the part that binds with the lysosome is ultimately degraded, exocytosis is the process by which the substance made by the component that binds to the plasma membrane is released into the extracellular area. The substances released outside the cells eventually become exosomes [16, 17].

## 3 Factors Affecting the Secretion of Exosomes

Exosome biogenesis is tightly controlled by a number of proteins and their protein systems [3]. Any problem in the middle link may affect the secretion of exosomes. For example, in the first step, after the extracellular source material enters the cell, it hinders the extracellular material from reaching the extracellular lipid membrane, thus preventing the expression of cell surface proteins and the production of ESEs. The process of forming exosomes for processing and maturation naturally cannot run. In the second step, after the formation of ESE, it continuously matures under the action of intracellular TGN, forming late‐sorting endosomes (LSE). When TGN fails to function or changes in function, the final process also stays in the ESE stage. The key step in the third step is the formation of MVB and intraluminal vesicles (ILVs), and the factors that affect the formation and maturation of ILVs are also key factors affecting the third step. The main mechanism governing ILVs production is a protein termed endosomal sorting complex (ESCRT), demonstrating that functional ESCRT‐related proteins are necessary for the endosomal pathway's generation of exosomes [18, 19]. A crucial molecule that influences the synthesis of exosomes [20], programmed cell death 101 interacting protein (Alix) is a supporting element of the ESCRT process. Second, it induces the degradation of MVB by increasing its binding to lysosomes or autophagosomes, thereby reducing the number of exosomes secreted [3, 21]. In addition, Hunger and autophagy inducers such as rapamycin can also induce the degradation of MVB by promoting the binding of MVB to autophagosomes, thereby achieving the goal of reducing the secretion of exosomes [22]. The fourth and final step is when the exosomes have matured. The last two processes before exosome release are docking and fusion, which are regulated by the GTP enzyme homolog Ras‐related (Ral‐1) [23]. The Ras‐related GTP enzyme homolog (Ral‐1) influences the release of exosomes, which are then followed by docking and fusion. MVB is coupled with SNARE proteins on the cell membrane before being fused into the plasma membrane [24]. Ultimately, these functioning exosomes are released into the environment outside of cells by exocytosis (Table 1).

**TABLE 1:** Functions of different types of vesicles.

Vesicles types	Function
Extracellular vesicle (EV)	A collective term for all vesicles
Early sorting endosomes (ESEs)	Produced by teething vesicles that form mature sorting endosomes in the presence of TGNs
Multivesicular bodies (MVBs)	Release of exosomes after fusion with the plasma membrane
Late‐sorting endosomes (LSEs)	Early sorting of mature bodies of endosomes
Intraluminal vesicles (ILVs)	Exosomes predecessors
Exosomes	Diameter 30–200 nm, average 100 nm, small vesicles with multiple functions

## 4 Exosome Isolation and Identification

### 4.1 Exosomes Separation

Exosomes are present in all physiological fluids, including blood, urine, lymph, bile, saliva, milk, and amniotic fluid, throughout the body [25]. Its distinct size, mass density, shape, charge, and antigen exposure are only a few of its physical and chemical characteristics [26]. Based on its inherent physicochemical properties, numerous methods have been developed for the separation of exosomes, including volume exclusion chromatography, magnetically bead immunity and affinity, ultrafiltration, heparin affinity methods, and centrifugation (including ultracentrifugation and density gradient centrifugation). Ultracentrifugation, the most straightforward technique, has both its pros and cons. Although the technology for separating exosomes has been widely developed and applied, it is still difficult to obtain completely purified exosomes.

### 4.2 Identification of Exosomes

After the separation of exosomes, identification techniques are used to identify the obtained exosomes, which are usually described based on their size, density, and markers [10]. Dynamic light scattering (DLS), nanoparticle tracking analysis (NTA), transmission electron microscopy (TEM), freezing electron microscopy, scanning electron microscopy (SEM), and atomic force microscopy (AFM) are often employed techniques for finding exosomes. Exosome size and distribution can be assessed with DLS and NTA [27]. The combination of TEM and immunogold staining technology can provide structural details of exosomes [28]. The morphology of exosomes can also be described by freezing electron microscopy. But compared with TEM, it seems that its function is far less powerful than the latter. The size, shape, and integrity of exosomes may be assessed using SEM and AFM together [29]. Additionally, Western blotting, the enzyme‐linked immunosorbent assay (ELISA), and the photosensitizer magnetic bead detection system (ExoScreen) can be used to evaluate the purity and efficacy of exosome enrichment [30]. The potential of flow cytometry for both qualitative and quantitative analyses of exosomes has been shown in recent studies [31]. However, its drawbacks are also obvious. The lower measurement limit of this technology is 100 nm, and it cannot distinguish smaller exosomes, resulting in poor accuracy and resolution. So, based on the physical and chemical characteristics of the needed exosomes, one may combine two to three identification methods, and the accuracy may be greater than with only one identification approach.

## 5 Tumor Microenvironment

**FIGURE 3:**
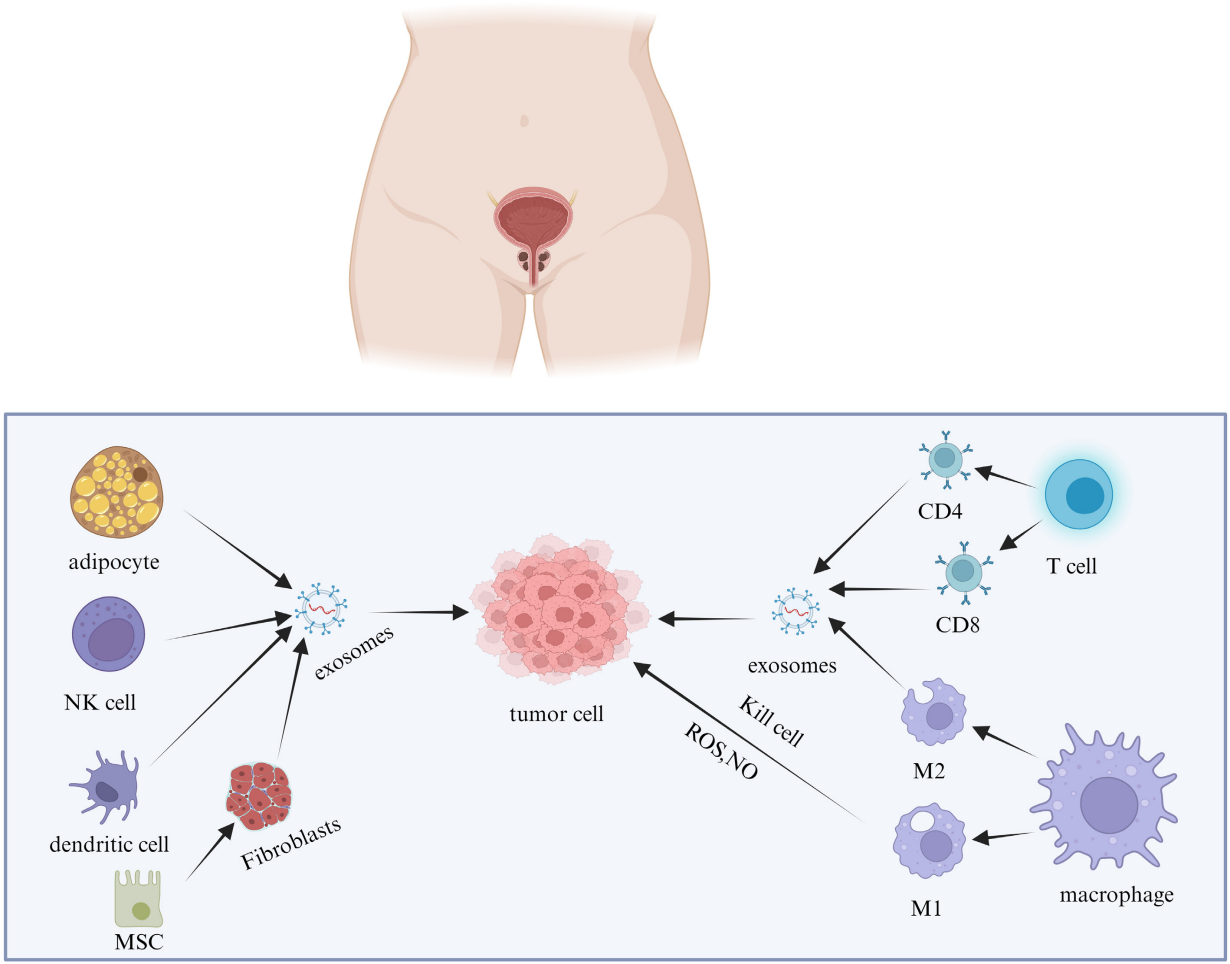
In TME, the influence of exosomes from different cell sources on tumor cells. Fat cells secrete various effectors and exosomes to promote cancer cell metastasis by altering the metabolism of cancer cells. Macrophages polarize toward M2 after receiving exogenous stimulation, and M2 macrophages secrete various cell mediators, including exosomes, to promote tumor progression. DC cells stimulated by foreign antigens can directly present antigens to T cells. In addition, DC cells can promote the expression of CD40, CD70, CD80, and CD86 molecules. T lymphocytes can differentiate into different immune cells such as CD4^+^, CD8^+^, and Treg cells, which have effects on tumor cells. NK cells can directly secrete cytotoxic particles such as perforin and granzyme to inhibit tumor growth.

Tumor cells, immune system infiltrating cells (macrophages, dendritic cells, and lymphocytes), cancer‐related stromal cells (cancer‐associated fibroblasts, CAFs), endothelial cells, lipids, extracellular matrix (ECM), and signaling molecules comprise the complex system known as the tumor microenvironment (TME) [32, 33]. Because TME is important for drug resistance, tumor immunosuppression, and targeted metastasis, it has garnered a lot of attention [34, 35, 36]. The concept of the tumor immune microenvironment (TIME) has a strong correlation to the prognosis of those suffering from tumors [37]. TIME contains various immune cell populations, with bone marrow cells and lymphocytes accounting for the majority [38, 39]. The immune response in TME is primarily controlled by TIME, but it also depends on the makeup and activity of invading immune cells as well as a number of related influencing variables, including the expression of immunological checkpoint molecules on cell surfaces and alterations in associated matrices. Both TME and TIME promote the progression of tumor cells through the presence of exosomes. Therefore, exosomes play an important role in TME. Next, we will mainly introduce the impact of several exosomes from different cell sources in TME on cancer progression (Figure 3).

## 6 The Impact of Exosomes Derived From Distinct Cell Sources on the Advancement of PCa Exhibits Variability

### 6.1 Adipocyte‐Derived Exosomes

Obesity has been linked in certain studies to worse outcomes from therapy, raised tumor invasiveness, and a higher risk of mortality in PCa patients [40, 41]. Especially relevant for patients with malignant recurrence of PCa and those who did not undergo postoperative radiotherapy and chemotherapy [42, 43, 44]. Adipose cells can release a variety of effectors, including exosomes, which play essential roles in paracrine and endocrine processes of local or systemic metabolic reactions. Exosomes generated from adipocytes may serve as a channel of communication between adipocytes and cancer cells. Fat vesicles play a crucial role in the development of cancer because they transport fatty acids, biological enzymes, protein‐degrading enzymes, metabolites, and other noncoding RNAs to cancer cells [45, 46, 47, 48]. Obesity not only increases the migration rate of preadipocytes in white adipose tissue but also mediates the progression of PCa [49]. It can also promote cancer cell proliferation and immune escape, which creates favorable conditions for creating a microenvironment for PCa progression [50]. Adipocytes' exosomes, in addition, cause PCa cells to proliferate and significantly augment their capacity for migration and invasion. This could be as a result of adipose exosomes' ability to speed up glycolysis processes, which in turn increases PCa cells' uptake of glucose, release of lactate, and synthesis of ATP [51]. Therefore, obese men with PCa have a worse prognosis than thin men.

### 6.2 Exosomes Derived From Macrophages

Numerous studies have shown that TME serves as a space for the survival and development of tumor cells, and macrophages play an immeasurable role in the TME. Tumor‐related macrophages, often referred to as M1 and M2 polarized macrophages, are a common form of macrophage seen in TMEs [52].

Modifying macrophage polarization toward M2 can accelerate tumor growth and contribute to therapeutic treatment resistance [53, 54]. This is mainly because when macrophages undergo M2 polarization, they can secrete proangiogenic factors and various cytokine mediators to promote tumor cell growth, invasion, and tumor‐related angiogenesis. A recent study that activated the STAT3 signaling pathway in PCa revealed the relationship between exosomes and macrophage polarization [55]. The expression of miRNA‐155‐3p, activated by IL‐6 and thus inducing autophagy, is augmented by exosomes containing high concentrations of both IL‐6 and miRNA‐155‐3p. Moreover, STAT3 activation is also a result of IL‐6. Due to the positive feedback pathway, it promotes the occurrence of tumors [56]. In addition, a study has revealed that exosomal RNF157 mRNA secreted by PCa cells can accelerate PCa progression by destabilizing HDAC1 protein to induce M2 polarization in macrophages by co‐culturing PCa cell lines with macrophages after transfection with RNF157 [57]. A recent study found that the endoplasmic reticulum of tumor cells in the TME was stressed under the influence of external factor stimuli (hypoxia, ischemia, and low pH), and that PCa cells released more exosomes to be taken up by macrophages under the effect of endoplasmic reticulum stress, affecting the M2 polarization of macrophages, thereby allowing tumor cells to evade immune surveillance and promoting tumor spread and metastasis [58].

Unlike M2 polarization, M1‐type macrophages have antitumor effects, which are able to distinguish between tumor cells and normal cells, and ultimately kill tumor cells by recognizing them. There are two different mechanisms of action for the tumor‐killing effects of M1‐type macrophages, one of which is the release of tumor‐killing molecules, such as ROS and NO, by M1‐type macrophages, which produce cytotoxic effects on tumor cells. In addition, macrophages can increase the secretion of inducible nitric oxide synthase, cell adhesion molecules, and so forth under INF‐γ stimulation, thus enhancing their tumor cell killing effect. Some scholars have used tumor cell–derived exosomes transfected with miR‐155 or miR‐125b2 to reprogram M2‐type macrophages to M1‐type macrophages in the TME, and achieve tumor cell killing through reprogramming [59].

### 6.3 Exosomes Derived From Dendritic Cells

DCs, a key antigen‐presenting cell (APC), are essential for tumor immunotherapy [60]. There are two main stages in their development: immature and mature [61]. The mucosal surface is home to the majority of immature DCs, and solid organs and skin act as sentinels to detect antigens. Although they are migratory, immature DCs do not release pro‐inflammatory substances. After consuming antigens, immature DCs move them to secondary lymphoid organs where they are presented to helper or effector T cells, which triggers specific CTL responses [62]. However, the migration effect of mature DCs is stronger than that of immature DCs. DCs, which are mature and have been reported to not only stimulate the expression of co‐stimulatory molecules such as CD40, CD70, CD80, and CD86, but also pro‐inflammatory cytokines and chemokines [63, 64], can directly present antigens to T cells and induce immune responses—a process known as T‐cell restimulation [65]. As an alternative, it can attach itself to APCs, process via the APC endosome route, and then return to the DC cell surface to be presented to T cells. Second, tumor cells have the ability to absorb DC‐derived exosomes, which then change into more potent immunological targets for effector immune cells [66]. Therefore, exosomes derived from DC can enhance the immune response of tumor cells through various pathways, thereby achieving the goal of inhibiting tumor growth.

### 6.4 Exosomes Derived From Natural Killer Cells

Natural killer (NK) cells are the third type of lymphocytes that are a part of the innate immune system, following T and B cells. As natural “killers,” NK cells have the ability to respond swiftly in the absence of antigen stimulation and deliver broad‐spectrum lethal effects. Moreover, NK cells have the power to regulate immune responses, especially T‐cell activity. NK cells may promote T‐cell differentiation, proliferation, and cytokine production [67]. The joint action of T lymphocytes and NK cells on the production of soluble factors and APCs affects these effects. NK cells have several primary ways by which they function as the body's first line of defense against the removal of malignancies. First, secrete cytotoxic particles such as perforin and granzyme to induce cell apoptosis. Second, binding to the corresponding receptors of tumor necrosis factor (TNF) superfamily members induces cell apoptosis. Subsequently, cytokines, growth factors, and chemokines are secreted in abundance by it, while macrophages, DC cells, and T cells collaborate to form immune systems. The presence of hazardous chemicals in NK cell–derived exosomes is the mechanism by which they exhibit antitumor actions [68]. For example, Fas ligand (Fas‐L) and perforin can induce tumor cell apoptosis [69]. It is interesting to note that hypoxia is a feature shared by the majority of solid tumors and can accelerate tumor growth. A recent study has demonstrated that hypoxia can induce NK cell–derived exosome secretion, augment the expression of cytotoxic molecules, induce tumor cell apoptosis, impede tumor cell migration, and accomplish the objective of treating cancer.

### 6.5 T‐Lymphocyte–Derived Exosomes

T lymphocytes, also called T cells, are pluripotent stem cells that are derived from bone marrow. These progenitors move to the thymus during the embryonic and early phases of human life, where they develop into immunocompetent T cells via thymic hormone induction [70]. External stimuli have the ability to generate T‐lymphocyte exosomes, which raise intracellular Ca^2+^ concentration and encourage secretion [71]. Exosomes produced by human T cells that have been activated are known to elicit activation‐induced cell death through the production of Fas‐L and APO ligand [72]. T‐cell–derived exosomes can interact with other immune cells to affect target cells and can also establish the ideal microenvironment for paracrine and autocrine immune system functions [73].

### 6.6 CD4 ^+^ Cell–Derived Exosomes

Exosomes from CD4^+^ T cells can boost the antitumor response of CD8^+^ T cells. These cells can also create cytokines and collaborate with other cells such as NKs, macrophages, and CD8^+^ T cells [74]. Exosomes produced by CD4^+^ T cells have a role in the control of humoral immunity in addition to influencing cellular immunity [75]. In a research, mice that had received the Hepatitis B surface antigen (HBsAg), vaccination responded to CD4^+^ T‐cell–derived exosomes more strongly than control mice, suggesting that the synergistic action of exosomes on HBsAb may help inhibit hepatocellular carcinoma [76]. So, through humoral immune control, may exosomes produced by CD4^+^ cells also prevent the spread of PCa cells? This needs more experimental confirmation.

### 6.7 Exosomes Derived From CD8 ^+^ Cells

White blood cells called CTLs have the ability to express a variety of cytokines that specifically destroy target cells, such as CD8^+^ T cells. They are a crucial line of defense for protection against viruses and tumors [77]. Further evidence suggests that CD8^+^T‐cell–produced exosomes may facilitate immune cell–tumor cell contact, hence inhibiting tumor progression. Tumor antigen–stimulated CD8^+^ cells produce exosomes, which stimulate low‐affinity CD8 T cells to participate in the process of eliminating tumors [78, 79]. Granzymes and perforin are found in the exosomes produced by CD8^+^ T cells, and they can be delivered directly when CTL fuses with the target cell membrane or enters the cell by endocytosis [80]. Exosomes are often released by CTL to help destroy target cells when they come into contact with them [81]. In addition to inhibiting antigen‐specific DC‐mediated CD8^+^ CTL responses by acting on target cells through membrane fusion and endocytosis, CD8^+^ T‐cell exosomes can also, in an antigen‐dependent manner, suppress antitumor immunity [82].

### 6.8 Exosomes Derived From Treg Cells

A further group of CD4^+^ T cells called regulatory T cells (Tregs) help tumor cells elude immune monitoring and govern the body's immune response in general [83]. Exosomes generated from Treg thus dramatically prevent the immune system. Different subtypes of Treg shut down unconventional immune cells in the microenvironment, including macrophages, DC, NK T cells, CD4^+^ T cells, CD8+ T cells, and B cells. According to research, CD4 and CD8 T lymphocytes' activation and growth are predominantly suppressed by Treg immunosuppression [84]. Furthermore, M1 macrophage marker expression is inhibited while M2 macrophage marker expression is promoted by Treg‐derived exosomes [85]. M2 macrophages can immunosuppress and promote tumors [86]. Therefore, Treg‐derived exosomes can not only inhibit various immune cell activities but also promote tumor progression by polarizing macrophages toward M2.

### 6.9 Exosomes Derived From Cancer‐Related Fibroblasts

A vital part of the TME is played by CAFs [87]. It can promote tumor progression through various mechanisms [88, 89, 90]. In addition, CAF can also inhibit immune responses in the TME by inducing M2 macrophage polarization or directly inhibiting the function of immune cells, thereby promoting tumor progression [91, 92, 93]. Additionally, CAF can influence other cells in the TME by secreting growth factors, with exosomes possibly having the most influence. Other growth factors that CAF might secrete include cytokines and growth factors. There is a lot of evidence to suggest that exosomes generated from CAF could interact with tumor cells [94]. Recent studies have revealed that mesenchymal stem cells (MSCs) also serve as CAF progenitors [95, 96]. However, the effect of stimulating MSC transdifferentiation varies depending on the cancer. In PCa, MSCs activate TGF secreted by tumor cells and stromal cells—β1 and C—X—C chemokine ligand (CXCL) 16 transdifferentiate into CAF [97, 98].

### 6.10 Exosomes Derived From Tumor Cells

Exosomes generated from tumors have an association with PCa's development and distal metastasis. The process of short‐ and long‐range communication between cancer cells, the TME, and the sites of metastasis is mediated by exosome‐mediated extracellular communication. Exosomes from tumors have a significant role or provide signals for sustaining intercellular activity [99, 100, 101]. The cellular breakdown of collagen and fibronectin by tumor exosomes, whereby alter integrin‐mediated cell adhesion, boosts tumor cell invasion and metastasis [102, 103]. According to studies, the Src protein is found in the exosomes of PCa cells and stimulates focal adhesion kinase through integrins to encourage angiogenesis and metastasis [104].

Studies show that absorbing PCa by bone marrow cells both in vitro and in vivo results in decreased expression of bone marrow platelet‐1, enhanced osteoclast development, and NF‐activation of B–cell signal transduction [105]. The author of a different study gave us insight into how phospholipase D2 increases exosome release in PCa cell line models and how it may increase osteoblast activity, which in turn enhances PCa bone metastases [106].

The initiation of metastasis is significantly aided by epithelial‐to‐mesenchymal transition (EMT), and tumor‐derived exosomes can activate signal transduction in either paracrine or autocrine forms. For instance, DU145 and PC3 cells' PCa‐derived exosomes generate TGF‐β, capable of initiating this pathway. In addition, the SMAD signaling pathway promotes tumor growth, triggers the conversion of fibroblasts into myofibroblasts, and suppresses the immune system [107, 108]. Similar to how exosomes from LNCaP and PC3 cells include integrin subunits 3 (ITGA3) and 1 (ITGB1), these proteins aid in the migration and invasion of these tumors [109].

Additionally, PCa cells are suitable for lymphatic metastasis pathways in addition to the traditional methods of peripheral infiltration and blood metastasis. This is primarily because tumor cells infiltrate lymph nodes in order to reach their target sites, which in turn promotes the invasive phenotype in PCa [110, 111, 112]. Although the mechanism underlying the lymphatic metastasis pathway of tumor cells is still unclear, one possible mediator leading to distant lymph node metastasis is exosomes [113, 114, 115]. The presence of TNF‐α in DU145, PC3, LNCaP‐SF, and LNCaP PCa cell lines was demonstrated by a study. This TNF‐α is known to promote metastasis of the PCa lymph node through activation of the C—C motif's chemokine ligand 21/C—C chemokine receptor 7 axis—specifically, exosomes [116, 117].

Additionally, tumor exosomes can increase the tumor cells' tolerance to chemotherapy treatments, increase their sensitivity to it, and promote the development of malignant cells. Previous research has linked exosomes to enzalutamide resistance, and it has been demonstrated that preventing exosome release can partially restore the sensitivity of PCa cells resistant to enzalutamide [118]. Second, in DU145, 22Rv1, and LNCaP [119], docetaxel‐sensitive PCa cells and exosomes contribute to increased resistance to the drug. Furthermore, exosomes generated from tumor cells have the ability to transfer miRNA, circRNA, adipokines, and inflammatory components to adipocytes, hence affecting the growth of adipose tissue and substance release. This can further transform fat into adipocytes associated with tumors. Adipose stem cells can undergo a prostate tumors‐like transformation when H‐ras, miR‐125b, miR‐155, and GTP enzyme compounds are secreted by tumor exosomes, for instance [120]. Tumor cells thrive in this environment because of the two positive feedback connection. According to the previously mentioned data, tumor‐derived exosomes play a critical role in the development, expansion, and metastasis of PCa (Table 2).

**TABLE 2:** The role of tumor cell–derived exosomes in prostate tumors.

Origin of exosomes	Mechanism	Function	References
Tumor cell derived	Breakdown of collagen and fibronectin and alteration of cell adhesion	Promote tumor cell invasion and metastasis	[100, 101]
	Src protein mediates integrin activation of focal adhesion kinase	Promote angiogenesis in tumor cells	[102]
	Phospholipase D2 enhances the activity of citrophagocytes	Enhancement of bone metastasis of tumor cells	[104]
	TGF‐β initiates the epithelial–mesenchymal transition (EMT) pathway	Promote the metastasis of tumor cells	[105, 106]
	TNF‐α activates the C—C motif of the chemokine ligand 21/C—C chemokine receptor 7 axis	Promote lymph node metastasis of tumor cells	[114, 115]
	Enhancing tolerance to tumor chemotherapy drugs	Promote tumor cell invasion, metastasis	[116]
	Conversion of adipocytes to tumor‐associated adipocytes	Promote tumor cell invasion and metastasis	[118]

### 6.11 Exosomes' Potential for PCa Diagnostics

Currently, PSA is the most commonly used biomarker for PCa by clinicians. However, many men with elevated PSA levels do not have PCa, and while PSA is organ specific, it is not cancer specific, and in the presence of disease that destroys the epithelial cells of the basement membrane of the prostate (e.g., prostatitis, benign prostatic hyperplasia, prostate biopsy, and surgery), the level of PSA in the bloodstream is usually elevated as well [9]. In addition to this, because of the low sensitivity of PSA in diagnosing prostate tumors, the positive rate of PSA in routine testing is only 58.3%, especially in the early stage when the diagnosis of PCa does not effectively differentiate between PCa and BPH, and a prostate biopsy is needed to confirm the diagnosis [121]. Recently, some researchers analyzed miR‐141 levels in serum exosomes of PCa patients compared with patients with prostate hyperplasia (BPH) and healthy controls (PCa, BPH, and healthy controls *n* = 20) Finally, it was found that miR‐141 expression was significantly upregulated in the exosomes of PCa patients (3.85‐fold, *p* = 0.0007 and 4.06‐fold, *p* = 0.0005) [122, 123].

Exosomes might be important indicators for PCa early detection, individualized treatment plans, and long‐term patient prognosis [124]. Body fluids from individuals with PCa, such as saliva, urine, and blood, include exosomes that have been produced and display a variety of proteins on their surface, known as epitopes [125]. At present, the detection of exosomes is relatively complete, and different methods can be used to specifically detect exosomes in different body fluids. According to recent research, PCa exosomes are abundant in PSA, which is indicative of the properties of early PCa cells [126]. The strongest candidate for intercellular communication in PCa is extracellular RNA [127]. Through the collection and analysis of clinical samples, an author confirmed that plasma exosome miRNAs have a high diagnostic value for patients with PCa [128]. Exosomes are a rich source of proteins, glycoconjugates, and nucleic acids; they also play a role in several biological signaling pathways and mediate intercellular communication within the TME. Exosomes are secreted by a variety of cells and are found in the secretions of the prostate, urine, serum, saliva, and semen. In clinical use, they may represent a new generation of early tumor screening instruments.

### 6.12 Exosomes' Potential as a PCa Treatment

Exosomes are the perfect therapeutic delivery vehicle due to their stability, biocompatibility, permeability, low toxicity, and low immunogenicity [129]. By means of enhanced circulatory stability and permeability of the biological barrier, they can be utilized as effective carriers of chemotherapy to enhance the regulation of target tissues and organs [130]. Various secretions can be transported by exosomes to specific cells where they can take effect. For instance, mesenchymal stromal cells have the ability to package and deliver active medicines via secreted vesicles [131]. Targeted modification of exosomes to deliver azithromycin to tumor cells for therapeutic purposes [132]. Meanwhile, drug‐loaded exosomes can enhance tumor cell targeting and inhibit tumor growth [133]. By increasing their cytotoxicity, exosomes produced from tumor cells can be utilized as an efficient vehicle for paclitaxel against autologous PCa cells [134]. Exosomes serve as carriers for delivering therapeutic drugs to tumor cells, which not only effectively kill tumor cells but also minimize the side effects of drugs [135].

## 7 Conclusion

PCa is the most common solid tumor and in many cases, early‐stage tumors often go undetected, just by a single PSA test is not the only evidence to confirm the diagnosis, therefore, the development of a novel test is necessary. Exosomes, as a type of cellular vesicle, contain many different surface proteins that can be recognized on their surface. Not only that, exosomes present in body fluids throughout the body are considerably more compliant and convenient for patients compared to solid biopsy sampling. In addition, exosomes are highly stable in biological fluids, provide excellent protection against their loaded biomarkers, and can be used to differentiate between tumor risk, suboptimality, recurrence potential, and progression based on molecular content. Undoubtedly, biomarkers with these optimal properties offer great promise for effective screening, diagnostic, prognostic, and therapeutic detection of PCa in clinical applications.

In conclusion, exosomes are biological vesicles that cells release and have qualities including substance transport, signal transduction, minimal immunogenicity, and simple circulation. By moving different hazardous chemicals, they can facilitate the spread of cancer. Exosomes, as important tools for intercellular communication and transportation, mediate information exchange between cells by transmitting their secreted substances (RNA, proteins, etc.) to receptor cells and influencing their physiological functions. Exosomes provide rich biological information and are attractive liquid biopsy specimens with high application value. In addition to genetic molecules, exosome‐related proteins have also been widely studied as potential disease‐related biomarkers. As a result, exosomes have clear benefits in diagnosis and treatment, but they are not without limitations. Exosomes can be extracted from a variety of bodily fluids, but there is no procedure for identifying, grading, and describing exosomes. Furthermore, false‐positive or false‐negative results could arise from the exosomes' surface containing molecules that are identical to the target test chemical. In addition, differences in the collected exosomes and their content can also affect individual research results. Therefore, current nonstandard measurements hinder reliable comparisons between studies. Exosome proteins play a crucial role in detecting exosome‐mediated tumor occurrence and development. Although there are still many challenges and we may still have a long way to go to fundamentally change cancer diagnosis by stimulating the potential of extracellular biomarkers, it can indeed open up new treatment pathways for personalized diagnosis and precision medicine and may bring a promising future to cancer patients.

## Author Contributions

**Cong Huang:** conceptualization (equal); data curation (equal); formal analysis (equal); writing – original draft (equal); **Jialong Zhang:** writing – review and editing (equal); **Hongzhi Wang:** project administration (equal); resources (equal); software (equal); supervision (equal); **Chaozhao Liang:** conceptualization (equal); data curation (equal); formal analysis (equal); investigation (equal); supervision (equal).

## Conflicts of Interest

The authors declare no conflicts of interest.
